# Ancient diversity and geographical sub-structuring in African buffalo *Theileria parva* populations revealed through metagenetic analysis of antigen-encoding loci^☆^

**DOI:** 10.1016/j.ijpara.2017.10.006

**Published:** 2018-03

**Authors:** Johanneke D. Hemmink, Tatjana Sitt, Roger Pelle, Lin-Mari de Klerk-Lorist, Brian Shiels, Philip G. Toye, W. Ivan Morrison, William Weir

**Affiliations:** a The Roslin Institute, Royal (Dick) School of Veterinary Studies, University of Edinburgh, Easter Bush, Roslin, Midlothian EH25 9RG, UK; b The International Livestock Research Institute, PO Box 30709, Nairobi, Kenya; c Department of Agriculture, Forestry and Fisheries (DAFF), National Department of Agriculture, PO Box 12, Skukuza, Kruger National Park, 1350, South Africa; d Institute of Biodiversity Animal Health and Comparative Medicine, College of Medical, Veterinary and Life Sciences, University of Glasgow, Henry Wellcome Building, Garscube Campus, Bearsden Road, Glasgow G61 1QH, UK; e School of Veterinary Medicine, College of Medical, Veterinary and Life Sciences, University of Glasgow, Bearsden Road, Glasgow G61 1QH, UK

**Keywords:** *Theileria parva*, African buffalo, Genetic diversity, T cell antigens, Cattle, Vaccination

## Abstract

An infection and treatment protocol involving infection with a mixture of three parasite isolates and simultaneous treatment with oxytetracycline is currently used to vaccinate cattle against *Theileria parva*. While vaccination results in high levels of protection in some regions, little or no protection is observed in areas where animals are challenged predominantly by parasites of buffalo origin. A previous study involving sequencing of two antigen-encoding genes from a series of parasite isolates indicated that this is associated with greater antigenic diversity in buffalo-derived *T. parva*. The current study set out to extend these analyses by applying high-throughput sequencing to ex vivo samples from naturally infected buffalo to determine the extent of diversity in a set of antigen-encoding genes. Samples from two populations of buffalo, one in Kenya and the other in South Africa, were examined to investigate the effect of geographical distance on the nature of sequence diversity. The results revealed a number of significant findings. First, there was a variable degree of nucleotide sequence diversity in all gene segments examined, with the percentage of polymorphic nucleotides ranging from 10% to 69%. Second, large numbers of allelic variants of each gene were found in individual animals, indicating multiple infection events. Third, despite the observed diversity in nucleotide sequences, several of the gene products had highly conserved amino acid sequences, and thus represent potential candidates for vaccine development. Fourth, although compelling evidence for population differentiation between the Kenyan and South African *T. parva* parasites was identified, analysis of molecular variance for each gene revealed that the majority of the underlying nucleotide sequence polymorphism was common to both areas, indicating that much of this aspect of genetic variation in the parasite population arose prior to geographic separation.

## Highlights

• Buffalo-derived *Theileria parva* genotypes are highly diverse.
• Buffalo consistently exhibit a high multiplicity of infection.
• Several bovine CD8 T cell antigens are highly conserved at the amino acid level.
• Kenyan and South African parasite populations are geographically structured, but share ancient diversity.
• Data indicate that recombination plays a major role in generating diversity in field populations.

## Graphical abstract

**Figure:**
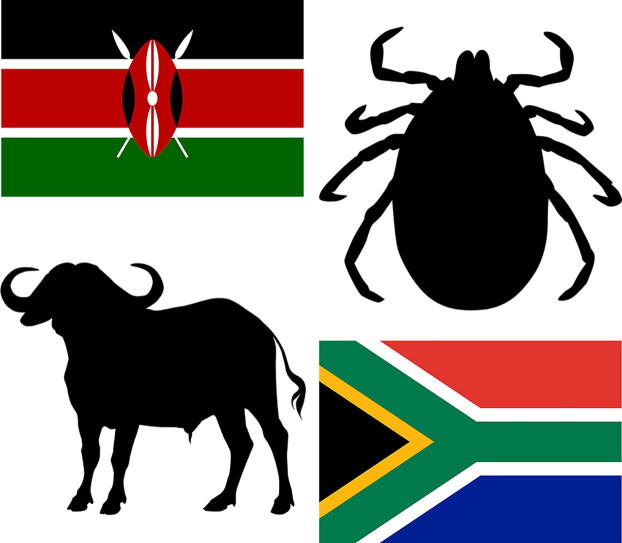

## 1 Introduction

Antigenic heterogeneity is a key feature of a number of important protozoan pathogens of animals and humans, including *Plasmodium* spp. (Kyes et al., 2001), *Eimeria tenella* (Blake et al., 2015) and *Theileria parva* (Morrison et al., 2015). In the latter two species, live parasites are used for vaccination and inclusion of more than one parasite genotype in the vaccines is required to provide protection in the field. However, until recently, the antigenic basis of the immunological heterogeneity of these parasites has been poorly understood.

The apicomplexan parasite *T. parva*, transmitted by the three-host tick *Rhipicephalus appendiculatus*, infects cattle and African buffalo (*Syncerus caffer*). It causes an acute, often fatal lymphoproliferative disease in cattle, known as East Coast fever (ECF), which is a major constraint to livestock production throughout a large part of eastern and southern Africa (Irvin and Morrison, 1987). The parasite infects lymphocytes, in which it develops to the multinucleate schizont stage, which induces activation and proliferation of infected cells (Dobbelaere and Rottenberg, 2003). By associating with the host cell spindle during mitosis, the parasite is able to divide at the same time as the host cell, resulting in maintenance of infection in the daughter cells (von Schubert et al., 2010). In cattle, this process leads to progressive expansion of the parasitised cell population and severe disease (Morrison et al., 1981). The subsequent intra-erythrocytic piroplasm stage causes little or no pathology, but is required for onward transmission by ticks. Animals that manage to recover from the acute phase of disease develop low level carrier infections which can persist for many months or years and are an important source of parasites for tick transmission (Kariuki et al., 1995, Skilton et al., 2002).

Although *T. parva* is able to infect and transform buffalo lymphocytes in the same manner as cattle cells, infections in buffalo are not associated with overt clinical signs. Most buffalo residing in ECF-endemic areas are infected with *T. parva* (Young et al., 1978) and hence they represent a reservoir for infection of cattle. Cattle that acquire infection from ticks that have fed on buffalo develop severe disease, but these parasites differentiate poorly to the tick-infective piroplasm stage in cattle and therefore are not transmitted or are transmitted at low efficiency by ticks to other cattle (Neitz, 1957, Schreuder et al., 1977, Grootenhuis et al., 1987, Mbizeni et al., 2013). Available data suggest that, as a consequence of this barrier, populations of *T. parva* found in buffalo differ genotypically from those maintained in cattle (Oura et al., 2011, Pelle et al., 2011). In South Africa, *T. parva* is currently confined to buffalo, cattle-maintained *T. parva* having been eradicated in the first half of the last century and transmission from buffalo to cattle controlled by strict separation of infected buffalo from cattle (Norval et al., 1992).

In the 1970s a method of vaccinating cattle against *T. parva* was developed, involving infection with *T. parva* sporozoites and simultaneous treatment with long-acting tetracycline (reviewed in Morrison and McKeever, 2006). Although immunisation of cattle with one parasite isolate resulted in solid long-lasting immunity to challenge with the same isolate, a proportion of immunised animals remained susceptible to challenge with other isolates (Radley et al., 1975a). Following a series of immunisation and challenge experiments, a combination of three parasite isolates was identified which generated immunity against experimental challenge with a range of *T. parva* isolates of cattle origin (Radley et al., 1975b) and against field challenge (Uilenberg et al., 1976). This mixture of parasites, known as the Muguga cocktail, is currently used to vaccinate against the disease in eastern Africa (Di Giulio et al., 2009).

Studies of immune cattle have indicated that parasite-specific CD8^+^ T cell responses play a key role in immunity and have shown that lack of cross-protection between parasite isolates is reflected by strain specificity of the CD8^+^ T cell responses (McKeever et al., 1994, Taracha et al., 1995a, Taracha et al., 1995b). Although the Muguga cocktail vaccine has been used successfully in some regions, there is evidence that it does not protect all animals against infections acquired by transmission from buffalo (Radley et al., 1979). Thus, two recent studies found that vaccinated cattle introduced into areas grazed solely or predominantly by buffalo showed no or low levels of protection against disease (Bishop et al., 2015, Sitt et al., 2015). These findings imply that *T. parva* parasite populations in buffalo harbour a greater antigenic diversity than those in cattle. Evidence to support this contention was provided by a study of the sequences of two genes (Tp1 and Tp2) which encode proteins recognised by CD8^+^ T cells from immune cattle, in infected cell lines isolated primarily from eastern Africa (Pelle et al., 2011). Over 30 allelic variants of each antigen were identified, and a large majority of these were found in isolates obtained from buffalo or from cattle that had grazed alongside buffalo, whereas only a small subset of the variants was detected in the isolates obtained from cattle grazed in the absence of buffalo (Pelle et al., 2011).

Given the evidence of genetic diversity in buffalo populations of *T. parva*, the current study set out to extend these analyses by applying a metagenetic, high-throughput sequencing approach to determine the extent of polymorphism in a panel of antigen-encoding genes. Samples from two geographically distant populations of buffalo, one in Kenya and the other in South Africa, were examined in order to investigate the influence of geographic separation on parasite genetic diversity. The results demonstrate extensive allelic diversity in all genes examined, both at the individual animal and population levels of the buffalo, but also reveal that some of the parasite antigen genes show a high level of conservation at the amino acid level. Comparison between the Kenyan and South African *T. parva* sequences indicates that much of the underlying genetic variation is found in both buffalo populations and is therefore likely to have been present in a common ancestor prior to geographical separation.

## 2 Materials and methods

### 2.1 Buffalo DNA

Samples of genomic DNA were obtained from single blood samples from eight African buffalo on the Ol Pejeta Conservancy in the Laikipia district of Kenya (Sitt et al., 2015) and from six buffalo in the Kruger National Park, South Africa. Ethical approval for the project was provided by the International Livestock Research Institute (ILRI), Kenya through its Institutional Animal Care and Use Committee (IACUC Approval 2011–11). Permission for the collection of samples from Ol Pejeta Conservancy was provided by the Kenya Wildlife Service, Kenya (KWS/BRM/5001) and from culled buffalo in the Kruger National Park by the South African National Parks, South Africa. DNA was extracted from the Kenyan buffalo samples using a DNeasy Blood and Tissue Kit (Qiagen, USA). The South African samples were provided by Dr. Nicholas Juleff, Pirbright Institute, UK. Further information on the origin of the buffalo, their sex and estimated age can be found in the National Center for Biotechnology Information, USA (NCBI) BioSample database under accession numbers 5712932–5712932.

### 2.2 Sequencing of 18S rRNA subunit

The presence of different species of *Theileria* and *Babesia* in the blood samples was investigated by sequencing a 375 bp segment of the 18S rRNA gene using Roche 454 amplicon sequencing technology as previously described (Bishop et al., 2015).

### 2.3 Sequencing *T. parva* antigen-encoding genes

A high-throughput multi-locus sequence typing system involving Roche 454 sequencing of PCR amplicons of selected *T. parva* genes was employed to identify allelic variation in the target genes. Amplicons of segments (292–492 bp) of six genes known to encode antigens capable of recognition by bovine CD8^+^ T cells (Tp1, Tp2, Tp4, Tp5, Tp6 and Tp10) (Graham et al., 2006, Hemmink et al., 2016) were generated as previously described (Hemmink et al., 2016). Regions containing known CD8^+^ T cell epitopes were selected. The genomic references of these genes, the gene-specific primer sequences and the PCR conditions used are reported elsewhere (Hemmink et al., 2016). For highly polymorphic loci such as Tp2, degenerate primers were selected to facilitate amplification from a diverse range of allelic sequences. In preliminary studies, these primers successfully generated PCR products from a panel of 32 genotypically diverse cloned *T. parva*-infected cell lines, which included 22 isolates from buffalo or from cattle grazed alongside buffalo. Primers had also been tested for *T. parva* specificity using DNA from other *Theileria* spp., namely *Theileria annulata*, *Theileria buffeli*, *Theileria taurotragi* and *Theileria* sp. (buffalo), as well as host DNA (Hemmink et al., 2016)*. Theileria* sp. (buffalo) is a recently identified species, closely related to *T. parva*, which has not yet been allocated a definitive species name. Four cloned cell lines confirmed to be infected with *T.* sp. (buffalo) by sequencing of 18S ribosomal subunit DNA were included in the panel. The final primers used only amplified *T. parva* DNA.

Amplicons were generated for each of the samples using fusion primers, comprising the gene-specific primer sequence, a Multiplex IDentifier (MID) and an adaptor sequence required for 454 processing of the amplicon, as previously described (Hemmink et al., 2016). PCR products were tested for purity and equimolar quantities of product were pooled from reactions with different templates but targeting the same gene. These pooled PCR products were submitted to the Centre for Genomics Research (CGR, University of Liverpool, UK) for Roche 454 sequencing.

### 2.4 Analysis of high throughput sequence data

Raw 454 sequencing data were processed using a custom bioinformatic pipeline as previously described (Hemmink et al., 2016). Briefly, groups of reads representing individual PCRs were identified and extracted from multiplexed sequence datasets on the basis of their MID tags and primer sequences. The raw reads for each gene in each single sample were then subjected to bioinformatic ‘noise’ reduction (shhh.flows and shhh.seqs) and a chimaera detection process, using algorithms implemented on the Mothur platform (Schloss et al., 2009). Three different algorithms were used in order to maximise the likelihood of chimaera detection, namely Perseus, Chimera Slayer and Uchime (Edgar et al., 2011, Haas et al., 2011, Quince et al., 2011). ‘Variants’ at a frequency below 0.4% of total reads or less than five reads for each target gene were removed from the dataset. Since sequence quality was reduced towards the end of the relatively long Tp2 reads, and forward and reverse reads could not be paired with confidence, only the first ∼240 bases of the forward reads of this gene were used for analyses.

### 2.5 Phylogenetic and population genetic analysis

The number of unique alleles per gene for each of the animals and heterozygosity per gene in each animal were determined using custom scripts in the bioinformatic pipeline. Heterozygosity was calculated using the formula: H_e_  = 1 − ΣPi^2^, where P is the proportion of allele i. Information on sequence polymorphism and nucleotide diversity (π) was obtained using DNASP v5 software (Librado and Rozas, 2009). Phylogenetic network analysis was performed using Splitstree (Huson and Bryant, 2006) with a ‘Neighbor Network’ being constructed for each gene. For the population analysis, an allele-sharing matrix (ASM) was constructed based on Jaccard’s similarity index (Jaccard, 1908) using a custom Perl script. Principal co-ordinate and analysis of molecular variance (AMOVA) analyses were undertaken using the Genalex plug-in for Excel (Peakall and Smouse, 2006), using distance matrixes constructed by MEGA7 (Kumar et al., 2016). The occurrence of nucleotide (nt) insertions and deletions in gene sequences were calculated relative to the Mugugua reference genome sequence (Gardner et al., 2005). Allelic datasets were screened for evidence of codons under positive purifying selection using the single-likelihood ancestor counting (SLAC) method implemented by Datamonkey (Pond and Frost, 2005).

## 3 Results

### 3.1 African buffalo are infected with a range of *Theileria* spp.

Buffalo DNA samples were first subjected to high-throughput sequencing of PCR amplicons of the parasite 18S rRNA gene to determine which *Theileria* spp. were present and to confirm the presence of *T. parva*. Although the PCR primer sequences employed were capable of detecting both *Theileria* and *Babesia* spp., only *Theileria* sequences were detected. The numbers of sequence reads corresponding to each *Theileria* sp. in each animal are shown in Table 1. Sequences corresponding to several species of *Theileria* were obtained, namely *T. parva*, *T.* sp. (buffalo), *Theileria velifera* and *T. velifera-*like, *Theileria mutans* and *T. mutans*-like, *Theileria buffeli* and *Theileria sinensis*–like. This range of species-specific sequences was similar to those reported previously in South Africa (Chaisi et al., 2011, Chaisi et al., 2013, Mans et al., 2011, Mans et al., 2016). *Theileria parva* was detected in all except one animal; however sequencing was only partially successful in this animal, yielding a total of just 23 sequence reads. In the remaining animals, *T. parva* sequences accounted for between 1.3% and 38.8% of the total *Theileria* sequence reads obtained. The presence of other closely related *Theileria* spp. in all the buffalo emphasises the importance of utilising primers with a high degree of species specificity to amplify target antigen-encoding genes.

**Table 1:** Number of 18s sequence reads corresponding to different *Theileria* spp. in African buffalo. Sequences in the *Theileria velifera* cluster from buffalo from the Kruger National Park were identical/similar to previously identified *T. velifera* and *T. velifera*-like sequences (JN572701 and JN572705). A novel *T. velifera-*like sequence was detected in each buffalo from Ol Pejeta Conservancy. Sequences in the *Theileria mutans* cluster from buffalo from the Kruger National Park were identical/similar to previously identified *T. mutans*-like 1 (FJ213585), *T. mutans*-like 2 (JN572696) and *T. mutans*-like 3 (JN572694). *Theileria mutans* cluster sequences detected in Ol Pejeta Conservancy buffalo were more similar to *Theileria* sp. (JN572700).

Sampling location	Animal ID	*T. parva*	*T. sp* (*Buffalo*)	*T. velifera* cluster	*T. mutans* cluster	*T. buffeli*	*T. sinensis*	Number of raw reads	Total number of good reads	Proportion of *T. parva* reads (%)
Kruger National Park, South Africa	SC01	27	89	116	118			922	350	7.7
	SC02		13		10			114	23	0.0
	SC03	6	357	97	13			664	473	1.3
	SC04	23	29	50	23			301	125	18.4
	SC05	149	455	370	72			2211	1046	14.2
	SC06	59	242	96	62			1642	459	12.9

Ol Pejeta Game Conservancy, Kenya	301	691	691	465			277	2346	2124	32.5
	302	187	246	111				1551	544	34.4
	303	463	869	134				1540	1466	31.6
	304	139	492	99	5			2167	735	18.9
	305	400	786	173	8			1483	1367	29.3
	306	8	44	11		39	25	243	127	6.3
	307	300	188	42			244	839	774	38.8
	308	56	71	37		542		984	706	7.9

### 3.2 Sequence diversity detected in antigen-encoding genes

PCR amplicons of segments of six *T. parva* genes that encode bovine CD8^+^ T cell target antigens were obtained from each of the 14 buffalo and subjected to high-throughput sequencing (NCBI Sequence Read Archive accession numbers SRX2060341–SRX2060423). Analyses of the sequence data demonstrated polymorphism in all six genes, both within individual animals and within each country. Overall, the number of variant nt sequences detected for each gene ranged from 36 alleles for Tp6 to 76 alleles for Tp4 (Table 2). The degree of allelic variation varied among the different genes. Consistent with previous studies, polymorphism was detected in over 60% of the nt residues in Tp2. This locus had the least associated reads of the six genes analysed and this may be related to lower efficiency of these PCR primers. The Tp4 gene showed variation in approximately 30% of the residues while the remaining genes varied at 9–12% of the residues. In addition, the set of Tp1 gene variants contained a complex indel compared with the genome reference sequence, as previously found (Pelle et al., 2011). A large insertion of variable length was found towards the end of the Tp1 gene segment, with the shortest sequence having a 36 nt deletion and the longest sequence having a 48 nt insertion relative to the Muguga reference sequence. In addition, a 3 bp insertion, as previously reported (Pelle et al., 2011), was detected in the Tp2 gene. The level of polymorphism for each gene was very similar in the Ol Pejeta Conservancy and Kruger National Park populations. Nucleotide diversity (π), defined as the average number of nucleotide differences per site between any two DNA sequences chosen randomly from the sample population, ranged from 0.0143 to 0.2241 for Ol Pejeta Conservancy and from 0.0122 to 0.2277 for the Kruger National Park. Many of the polymorphic nucleotide residues showed variation in both populations, however for each gene some positions only showed variation within one population.

**Table 2:** Summary of diversity found in *Theileria parva* antigen-encoding gene segments in populations of African buffalo from the Ol Pejeta Conservancy and the Kruger National Park.

Sampling location	Gene	Polymorphism in nucleotide sequence (polymorphic sites/all sites)^a^	Total No. of distinct nucleotide sequences	Nucleotide diversity (π)^a^	Polymorphism in amino acid sequence (polymorphic sites/all sites)	Total No. of predicted distinct protein sequence
Overall	Tp1	35/344 + ind	72	0.01447	20/118	66
	Tp2	168/243	72	0.22897	68/72	62
	Tp4	112/383	76	0.06415	2/40^b^	3
	Tp5	29/308	46	0.01799	0/69^b^	1
	Tp6	28/288	36	0.01730	1/96	2
	Tp10	30/278	42	0.02491	1/60^b^	2

Ol Pejeta Conservancy	Tp1	24/354 + ind	39	0.01467	15/118	31
	Tp2	158/243	33	0.22414	65/82	32
	Tp4	76/383	31	0.06047	2/40^b^	2
	Tp5	16/309	15	0.01655	0/69^b^	1
	Tp6	15/288	10	0.01426	1/96	2
	Tp10	22/278	26	0.02510	1/60^b^	2

Kruger National Park	Tp1	21/369 + ind	37	0.01217	12/119	36
	Tp2	159/243	44	0.22777	64/82	38
	Tp4	103/383	47	0.06341	2/40^b^	3
	Tp5	26/308	33	0.01784	0/69^b^	1
	Tp6	25/288	29	0.01639	0/96	1
	Tp10	23/278	17	0.02433	1/60^b^	2

a The insertion/deletion regions (ind) were excluded for the determination of the number of polymorphic residues in nucleotide sequences and π.

b The Tp4, Tp5 and Tp10 genes have predicted introns.

### 3.3 Multiplicity of infection

**Fig. 1:**
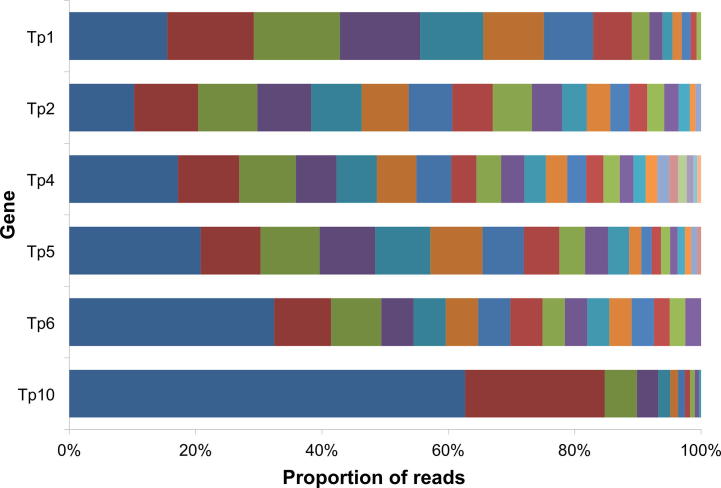
Example of allelic diversity at six *Theileria parva* antigen-encoding loci in a single buffalo. Proportion of different alleles of six *Theileria parva* gene segments (represented by different colours) detected in a single buffalo. The sequences were obtained using DNA from a buffalo (SC06) from the Kruger National Park, South Africa.

**Supplementary Fig. S1:**
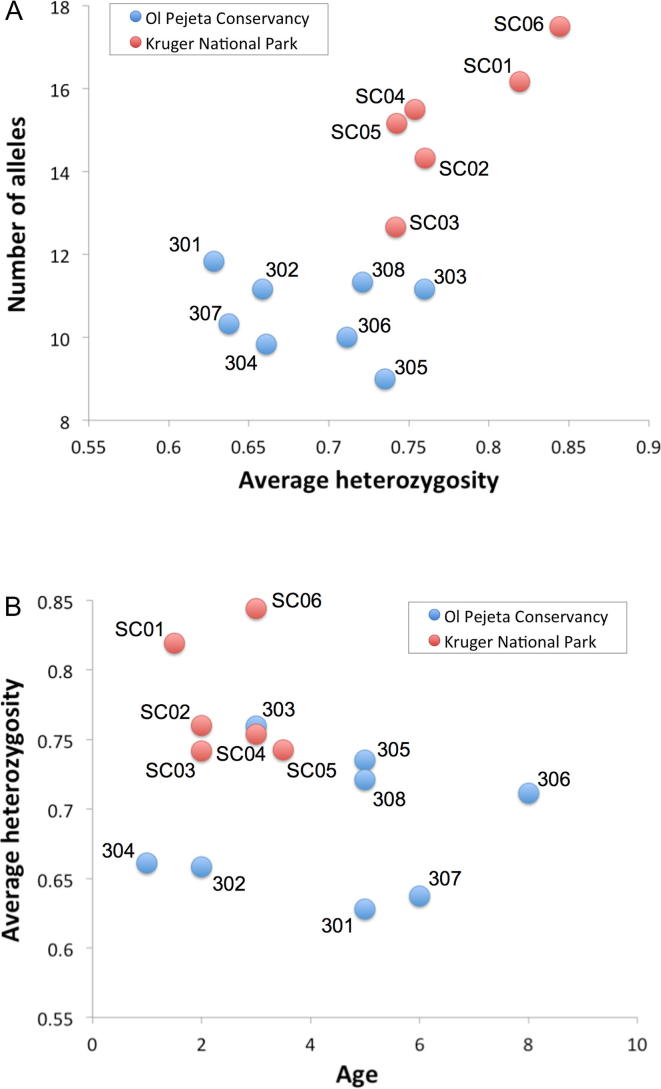
Within-buffalo *Theileria parva* diversity. (A) The average number of alleles and average heterozygosity per buffalo were compared and a positive correlation was identified (*r* = 0.72, *P* < 0.001). (B) The average heterozygosity per buffalo was compared with buffalo host age and a negative correlation was identified (*r* = −0.32, *P* = 0.26).

Samples from individual buffalo contained a large number of sequence variants of each gene. As an example, the number of sequence variants detected in the South African buffalo SC06, illustrated in Fig. 1, ranged from 24 (for Tp4) to 11 (for Tp10). To investigate the issue of multiplicity of infection, the average number of alleles per locus was compared with heterozygosity for each individual buffalo (Supplementary Table S1, Supplementary Fig. S1). These metrics, which each represent a different measurement of population diversity, were found to strongly correlate with one another (*r* = 0.72, Pearson correlation). Buffalo from the Kruger National Park had a higher number of alleles per locus and heterozygosity than those from Ol Pejeta Conservancy, and this is most evident in three genes (Tp4, Tp5 and Tp6). Buffalo from Ol Pejeta Conservancy had an average of 10.7 alleles per locus, whereas buffalo from the Kruger National Park had an average of 15.3, and this was statistically significant (*P* < 0.001, 2-tailed *T* test). Similarly, buffalo from Kruger National Park had a higher heterozygosity (0.78 versus 0.69; *P* < 0.01, 2-tailed *T* test). To investigate the relationship between host age and within-host parasite population diversity, the average heterozygosity was compared with estimated host age and a weak negative correlation was identified (*r* = −0.32, Pearson correlation) which was not statistically significant (Supplementary Table S1, Supplementary Fig. S1).

### 3.4 Phylogenetic analysis

**Fig. 2:**
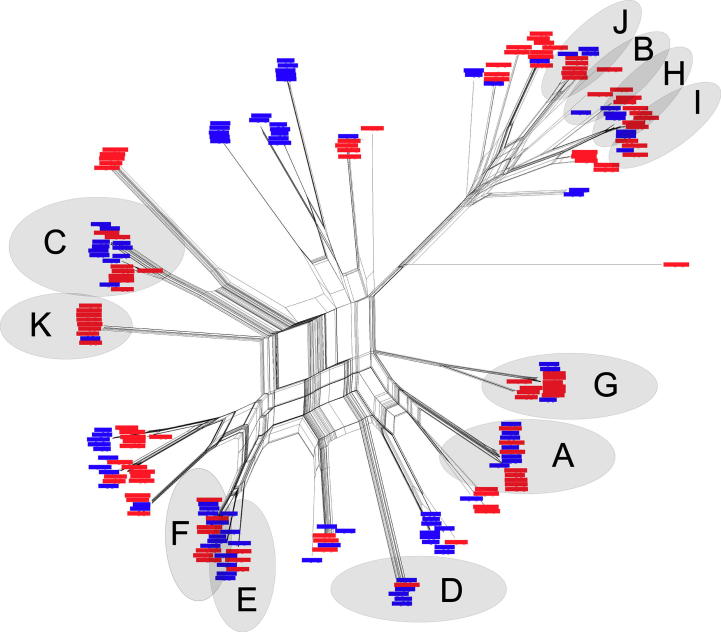
Phylogenetic network representing *Theileria parva* antigen-coding gene Tp2. A phylogenetic network was created based on allelic sequences of Tp2. Alleles identified from buffalo from Ol Pejeta Conservancy (Kenya) are shown in blue, while those from the Kruger National Park (South Africa) are shown in red. Eleven Tp2 haplotypes common to the Kenyan and South African parasite populations, based on epitope amino acid sequences, are illustrated (A–K).

**Supplementary Fig. S2:**
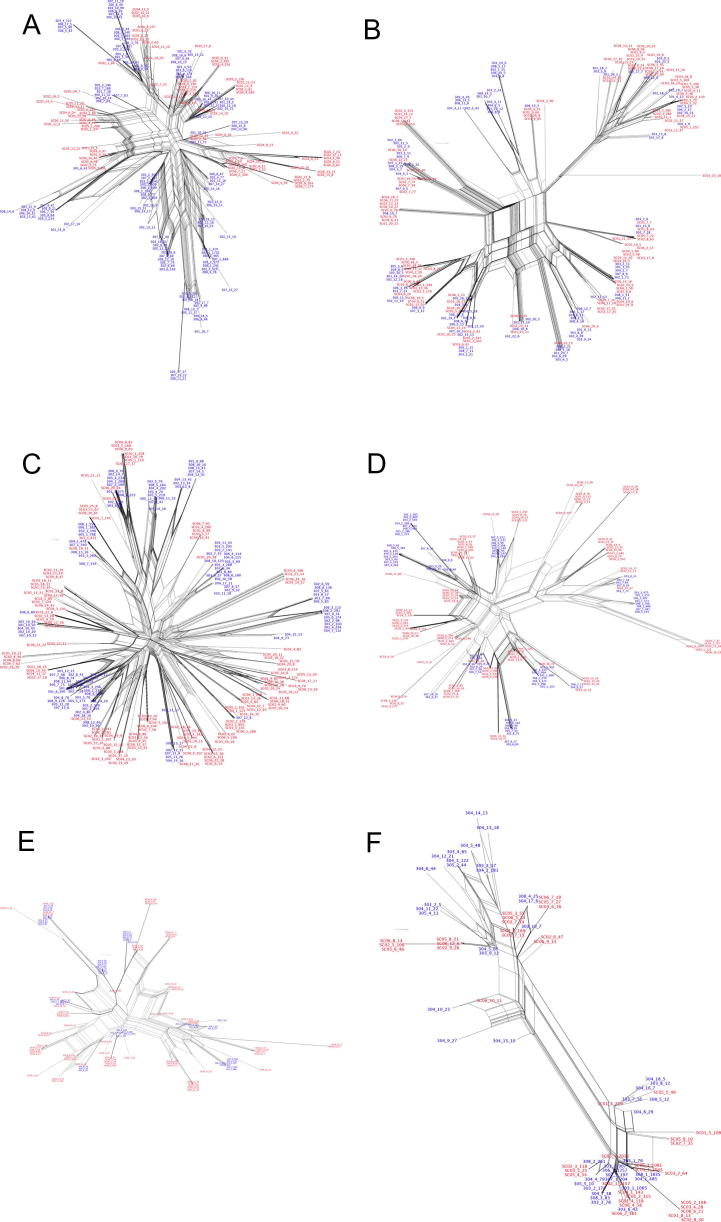
Phylogenetic networks for six *Theileria parva* antigen-encoding loci derived from buffalo. (A) Tp1; (B) Tp2; (C) Tp4; (D) Tp5; (E) Tp6 and (F) Tp10. Kruger National Park sequences are in red and Ol Pejeta Conservancy sequences are in blue.

To investigate the relationships among the different allelic (nt) sequences identified in this study, a series of phylogenetic networks was created (Supplementary Fig. S2). Each of these networks displays a high level of reticulation, strongly suggesting that extensive within-gene recombination is a feature in the evolution of these sequences. As an example, the phylogenetic network representing Tp2 is presented in Fig. 2. Similar to the other five genes, sequences derived from buffalo from both regions are interspersed throughout the network. Although several small clusters unique to each country can be discerned, the overall pattern is dominated by interspersion. This indicates that much of the underlying genetic variation at a nt level is present in both countries, suggesting that it predates geographical separation and is relatively ancient in origin. For example, in Tp10 (Supplementary Fig. S2F), two main allelic types can be discerned, with representative sequences obtained from samples from each country. Consistent with the results of the network analysis, Principal Co-ordinate Analysis (PCoA) of the nt sequences for each gene also failed to completely segregate the sequences based on geographical location (data not shown). These findings are supported by AMOVA, which showed that most of the diversity for each of the genes is found within populations (90–97%), with only a relative small proportion of the overall diversity partitioned between the populations (Supplementary Table S2). Out of the six genes, Tp1 showed the highest level of between-population diversity (10%) and this is supported by the topology of the phylogenetic network (Supplementary Fig. S2A), where several population-specific clusters of allelic sequences are evident.

### 3.5 Population differentiation

**Fig. 3:**
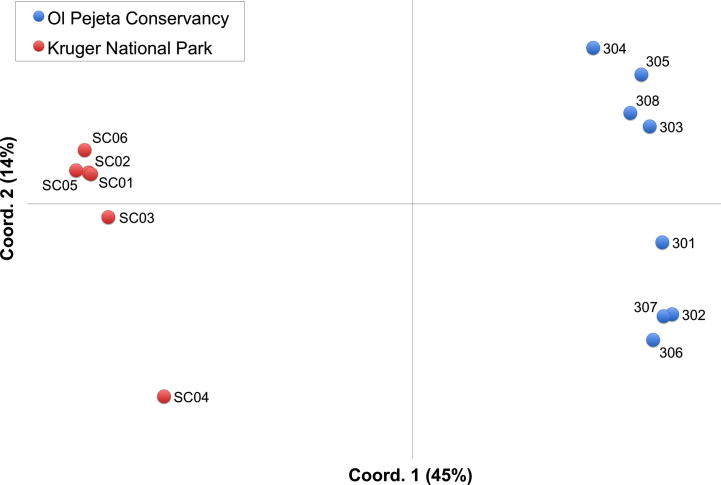
Genetic differentiation between *Theileria parva* populations in each buffalo. Principal co-ordinate analysis was performed to illustrate the genetic relationship between the parasite populations present within each buffalo. Allele-sharing matrices were generated for each of the six antigen genes based on Jaccard’s similarity index and an average was taken across all genes (Supplementary Table S3) which was used to perform the principal co-ordinate analysis. The proportion of the variation in the dataset explained by each axis is shown in parentheses.

The phylogenetic network analysis and AMOVA revealed a high level of diversity and a lack of overt geographical structure among allelic sequences of individual gene segments. However, as shown in Table2, most of the alleles (when compared across the sequenced segment of the six *T. parva* genes) were unique to either the Ol Pejeta Conservancy or the Kruger National Park animals. In order to further investigate whether quantifiable genetic differences exist between the parasite populations in the animals of each country, an allele-sharing approach was utilised. Using all six loci, a matrix of distances between each of the individual buffalo parasite populations was created based on the proportion of alleles shared by genotypes present in each buffalo (Supplementary Table S3). This matrix was used to perform PCoA, the results of which are presented in Fig. 3. The buffalo-derived parasite populations from each country clearly form separate clusters and these can be discriminated on the first axis of the PCoA. This strongly supports the contention that the parasite population is geographically sub-structured. This is reflected by the presence of polymorphic residues that are unique to either population, as shown in Supplementary Table S4.

### 3.6 The predicted amino acid sequences of some antigen-encoding genes are highly conserved

The results of analyses of the predicted amino acid sequences of the gene segments are summarised in Table 2. They reveal multiple allelic variants of the Tp1 and Tp2 gene products, similar to that reported previously for these two genes (Pelle et al., 2011). The variation included amino acid polymorphism in known CD8^+^ T cell epitopes within the proteins. By contrast, the translated amino acid sequences of the remaining four gene segments showed no or very limited diversity. This is reflected by relatively few non-synonymous nt substitutions in the coding region of these gene segments (Table 3). The amino acid sequences of the Tp5 gene segment were completely conserved and for the Tp4, Tp6 and Tp10 gene segments only three, two and two variable amino acid residues were detected, respectively (Table 2). One variant allele of the CD8^+^ T cell epitope identified in Tp4 was detected, which contained a single S to T substitution at position one in the epitope. It is of note that the sequenced segments of three of these genes, namely Tp4, Tp5 and Tp10, contained intron regions ranging from 98 to 261 nt in length. The number of nt changes was proportionally higher in the intron segments of the amplicons compared with the coding regions (Table 3). This finding, coupled with the observed conservation of the amino acid sequences of these genes, suggests they are broadly under purifying selection. This is supported by analysis of non-synonymous to synonymous substitution rates, with only a single codon in Tp5 under the influence of positive diversifying selection (*P* < 0.1), while Tp4, Tp5 and Tp10 showed evidence of purifying selection (*P* < 0.1) at 6, 3 and 1 codons, respectively (data not shown).

**Table 3:** Comparison of variable nucleotide residues in intron and exon regions of antigen-encoding *Theileria parva* gene segments.

Gene	Variable nucleotides in exon region(s)				Variable nucleotides in intron regions	
	Number	Percent	Non-syn^a^	Syn^a^	Number	Percent
Tp1	35/344	10.2%	22	13	–	–
Tp2	168/243	69.1%	156	12	–	–
Tp4	18/118	15.3%	3	15	92/265	35.9%
Tp5	10/208	4.8%	0	10	19/120	15.8%
Tp6	28/288	10.4%	1	27	–	–
Tp10	17/180	9.4%	1	16	13/98	13.2%

a Non-synonymous and synonymous nucleotide substitutions, respectively.

### 3.7 Similar variants of Tp1 and Tp2 CD8^+^ T cell epitopes are present in Kenyan and South African buffalo

**Supplementary Fig. S3:**
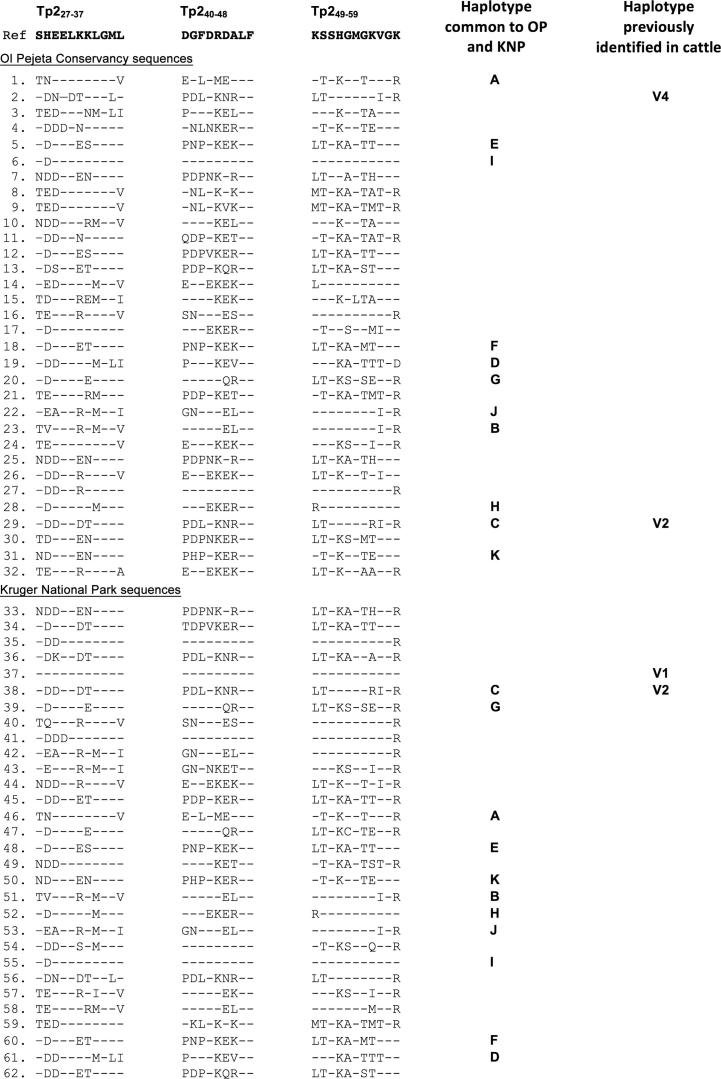
Amino acid sequences of three CD8^+^ T cell epitopes in 62 alleles of Tp2 identified in *Theileria parva* sequences obtained from Buffalo in Kenya and South Africa. Eleven haplotypes identified common to Ol Pejeta Conservancy (OP) and Kruger National Park (KNP) (A–K); three bovine haplotypes were identified (V1, V2, V3).

Previous analyses of Tp1 and Tp2 gene sequences identified three variants of a single Tp1 CD8^+^ T cell epitope and over 20 variants of a number of defined epitopes within the Tp2 protein. In both cases variation was associated with variable CD8^+^ T cell recognition (MacHugh et al., 2009, Connelley et al., 2011). Given the geographical separation of the two sets of buffalo examined in the current study, it was of interest to determine whether or not the epitope sequences in the two populations have diverged. Sequences for the Tp1_214–224_ epitope and the three epitopes (Tp2_27–37_, Tp2_40–48_ and Tp2_49–59_) in the sequenced segment of Tp2 were examined and compared with previously identified sequences. The same three variants of the Tp1_214–224_ epitope identified previously were represented in both the Ol Pejeta Conservancy and Kruger National Park sequences. The Tp2_27–37_, Tp2_40–48_ and Tp2_49–59_ epitopes showed extensive diversity in amino acid sequences, resulting in 43, 31 and 36 different epitope variants, respectively. The Tp2 epitope variants detected in the Kenyan and South African sequences are shown in Supplementary Fig. S3 and summarised in Table 4. Despite this marked overall diversity, a large proportion of the variants was common to both populations: thus, approximately half of the alleles of each epitope detected in the Kruger National Park samples were also found in the Ol Pejeta Conservancy samples. When amino acid allelic variation at the three Tp2 epitopes was investigated further, a total of only 51 Tp2 haplotypes was identified, much fewer than anticipated given the earlier evidence for recombination at this locus. Strikingly, 11 of these haplotypes were found to be common to both buffalo populations and this is illustrated in Fig. 2, where clusters of conserved haplotypes can be discerned. The South African parasites also included three Tp2 epitope haplotypes previously described in eastern African cattle isolates of *T. parva* (Pelle et al., 2011) (Supplementary Fig. S3).

**Table 4:** Numbers of distinct amino sequences detected for three CD8^+^ T cell epitopes in the *Theileria parva* Tp2 gene sequences obtained from Ol Pejeta Conservancy and Kruger National Park buffalo, and the number conserved between the two populations.

Parasite sequences	Number of unique epitope sequences		
	Tp2_27–37_	Tp2_40–48_	Tp2_49–59_
Overall^a^	43	31	36
Ol Pejeta Conservancy	28	25	27
Kruger National Park	27	20	23
Shared Ol Pejeta/Kruger	12	14	14

a The sequences of all three epitopes were conserved between the Kruger National Park and Ol Pejeta Conservancy in 11 of the sequences.

## 4 Discussion

Although there is compelling evidence that strain specificity of parasite-specific CD8^+^ T cell responses is a key determinant of incomplete cross-protection between different isolates of *T. parva* (reviewed in Morrison et al., 2015), there is limited information on the extent of antigenic diversity within field populations of the parasite. A previous study which applied conventional Sanger sequencing to examine single sequences of two T cell antigen-encoding genes (Tp1 and Tp2) from a series of cell lines isolated from diverse sources, predominantly in eastern Africa, demonstrated a high level of sequence diversity in parasites obtained from buffalo. The present study sought to investigate diversity in samples obtained directly from naturally infected buffalo by applying high-throughput sequencing to examine the sequences of an expanded set of six *T. parva* antigen-encoding genes. Two geographically distant groups of buffalo were examined to assess the potential effect of geographical isolation on sequence diversity.

All buffalo examined were found to be infected with multiple species of *Theileria* including *T. parva*, highlighting the need for species specificity of the PCR primers used to generate products for sequencing. The results of the sequence analyses revealed several important findings. First, nucleotide diversity was detected in all six gene segments examined, although the proportion of sites where polymorphism was evident varied, ranging from 9.7% to 69.1%. Second, large numbers of allelic variants of each gene were found in individual animals, indicating multiple infection events and/or multiple genotypes per infection. Third, despite the observed diversity in nucleotide sequences, several of the gene products had highly conserved amino acid sequences. Fourth, while there was evidence of genetic differentiation between parasite populations in the Kruger National Park and Ol Pejeta Conservancy, a substantial component of the sequence diversity, as shown by the AMOVA analysis, was shared between the two populations.

Although relatively small numbers of animals were examined, the extensive sequence diversity detected within individual animals enabled this set of six antigen genes to be used as genetic markers to assess the relationship between Kenyan and South African parasites. Based on these markers, the study demonstrated a high level of nucleotide diversity in both populations of buffalo *T. parva*, which varied from 0.0145 to 0.230. Moreover, the estimated average genetic ‘distance’ (as measured by the number of nt substitutions per site from averaging over all sequence pairs) between any two alleles in the Kruger National Park and between any two alleles within the Ol Pejeta Conservancy was very similar for all six genes examined. Phylogenetic network analyses and AMOVA of each of these genes indicated that most sequence diversity is common to both populations, with little of the variation partitioned between the Kruger National Park and the Ol Pejeta Conservancy. By sequencing antigen-encoding genes, rather than conserved ‘housekeeping’ genes, a highly polymorphic dataset was generated which was capable of detecting population differentiation. Of the six genes, Tp1 showed the greatest ability to discriminate between parasites from each location, with discernable but incomplete clustering of alleles from each area. By considering each buffalo as the host to a parasite population and performing a buffalo-buffalo comparison (Fig. 3), parasite meta-populations from each country can be clearly discerned. This approach effectively integrates the subtle location-associated variation at each locus and clearly demonstrates that the parasite population is geographically sub-structured. This is supported by the fact that many alleles were found to be ‘private’ to either the Ol Pejeta Conservancy or Kruger National Park buffalo, with some nucleotide changes being unique to parasites from either location (Supplementary Table S4).

Although buffalo-derived *T. parva* readily infect cattle, they differentiate poorly to the erythrocytic piroplasm stage and therefore are not readily transmitted by ticks to other cattle (Neitz, 1957, Schreuder et al., 1977, Maritim et al., 1992). Given the poor differentiation to tick-transmissible stages in cattle, transmission of these parasites to buffalo is also likely to be highly inefficient. Hence, geographical spread of *T. parva* by movement of infected cattle is unlikely to have made a significant contribution to geographical dissemination of buffalo *T. parva*. Spread would, therefore, be dependent on movement of infected buffalo. The *S. caffer* species of African Buffalo is found over a large area of eastern and southern Africa. Archaeological and genotyping data suggest that *S. caffer* evolved from an ancestor similar to the current western African buffalo (*Syncerus brachyceros*), as recently as 150,000–300,000 years ago, and subsequently disseminated southwards from eastern Africa (discussed in Smitz et al., 2013). Genotyping of local populations of buffalo has revealed a high overall level of genotypic diversity and a low level of genotypic differentiation between populations at a regional level, indicating extensive historical animal movement (Simonsen et al., 1998, Smitz et al., 2013, 2014). However, on a larger geographical scale, studies of 19 populations of buffalo over a large area of their southern habitat, extending from Zambia in the north to South Africa in the south, using mtDNA and microsatellite markers, identified genotypically distinct lineages, estimated to have diverged 6000–8400 years ago (Smitz et al., 2014). Although not strictly related to geographical distance, the two larger lineages tended to have a north/south distribution and the authors proposed that divergence had arisen historically as a result of local geographical and/or climatic barriers. More recently, human population expansion and associated erosion of habitat over the last century has resulted in progressive disruption of natural connectivity in the buffalo habitat and greater isolation of local buffalo populations. Hence, the two groups of buffalo examined in the current study, and consequently their parasite populations, are likely to have been separated for at least several centuries. As a consequence, the parasite populations would be genetically isolated from one another and this would explain the clear population differentiation identified, which may have arisen through genetic drift. The features of *T. parva* infections in buffalo, coupled with the genetic structure of the host and parasite populations, present opportunities for further more detailed studies to understand how the parasite has evolved in its natural host.

Previous analyses of Tp1 and Tp2 gene sequences from eastern African *T. parva* of cattle and buffalo origin provided evidence of positive selection for amino acid changes in the proteins, although it was unclear whether or not the selection was immune-based (Pelle et al., 2011). If indeed ongoing immune selection does influence sequence polymorphism, this might be most apparent in the T cell epitope regions of geographically separated parasite populations. However, this did not seem to be the case in sequences examined in the current study. The same three variants of the single epitope in Tp1 were present in both populations and, among the three highly polymorphic epitopes examined in Tp2, a large proportion of epitope variants were completely conserved between the Kenyan and South African buffalo parasites, and all but one of the small set of alleles found in cattle were also present in both populations. These findings further reinforce the evidence that a significant proportion of the variation in these genes is long-standing rather than being a consequence of recent or contemporary mutational changes.

Although the remaining four genes also showed allelic diversity in their nucleotide sequences, their translated amino acid sequences showed a remarkably high level of conservation and, in the case of Tp5, the sequence was completely conserved. Moreover, among the single T cell epitopes identified in Tp4, Tp5 and Tp10, only one variant of the epitope in Tp4, containing a single amino acid substitution, was found. This level of conservation at the amino acid level suggests that these gene sequences are under purifying selection and that conservation of the proteins may be required for parasite fitness, thus precluding any selection for coding changes. Buffalo have been shown to generate specific CD8^+^ T cell responses to *T. parva* (Baldwin et al., 1988), but in contrast to cattle (Graham et al., 2008), there is a lack of information on the antigenic specificity of these responses. Thus it is possible that these genes may not act as antigens in the context of a buffalo infection and epitopes defined in cattle would therefore not be expected to be under immune selection. It should be noted that even among cattle parasites, CD8^+^ T cell-associated immune selection may be relatively weak due to polymorphism in major histocompatibility complex (MHC) class I proteins among cattle populations in the field (McKeever, 2009).

A particularly striking finding in the current study was the detection of extensive allelic diversity in the *T. parva* gene sequences obtained from individual animals. This observation is inconsistent with infection having arisen from a single infective event, since previous studies of naturally infected tick populations have indicated that most ticks have one or only a few infected salivary gland acini (the sporozoites in each originating from a single parasite) (Moll et al., 1986, Gitau et al., 2000). Hence, the data indicate that buffalo repeatedly acquire infection from ticks in the field. Genotyping studies of *T. parva* in naturally infected cattle using satellite DNA markers have demonstrated an increase in the mean number of satellite DNA alleles with age, again suggesting repeated acquisition of infection (Oura et al., 2005). This is consistent with experimental evidence that induction of immunity to *T. parva* in cattle by an initial infection does not prevent establishment of infection following subsequent parasite challenge, even if animals are immune to clinical disease (Radley et al., 1975a).

The genotypic diversity of *T. parva* in infected buffalo is highly favourable for the occurrence of sexual recombination in the parasite, since most feeding ticks will imbibe mixed parasite genotypes, providing ample opportunity for zygote formation between divergent parasites. This is an ideal scenario for generating and maintaining enormous genotypic diversity in the parasite population and is consistent with evidence of extensive genetic exchange in field populations of *T. parva* in cattle (Oura et al., 2005). The results of the phylogenetic network analysis strongly indicate that historically this has included within-gene recombination, which is the most parsimonious explanation for the presence of extensive reticulation of the networks at each locus, causing conflicting phylogenies among segments of the same gene. For example, this can be observed in the most variable gene, Tp2 (Fig. 2), as well as between the two major allelic types of the highly conserved gene Tp10 (Supplementary Fig. S2F). The lack of obvious recombination between the three CD8^+^ T cell epitopes in Tp2 may reflect the absence of a cross-over breakpoint in this short segment of the Tp2 gene (99 nt). Nevertheless, the findings support the occurrence of recombination events within these genes over an extended period of time, before the populations diverged. This, however, does not preclude the occurrence of further within-population recombination events which may have taken place since the populations separated. Evidence for within-gene recombination events in *T. parva* has previously been generated, with a mosaic structure noted in the Polymorphic Immunodominant Molecule (PIM) (Geysen et al., 2004). It should be emphasised that the gene segments analysed in the present study each represent a very small fraction of the genome and the fact that recombination can be detected consistently over such limited regions suggests it is a very common feature throughout the parasite genome. A high frequency of recombination events has previously been documented in experimental crosses of *T. parva* (Katzer et al., 2011) with half of all cross-over breakpoints resulting in within-gene recombination events and the generation of novel alleles (Henson et al., 2012). In addition to the generation of allelic diversity at antigen-encoding loci as a consequence of within-gene recombination, extensive recombination in the parasite population will effectively result in the continual generation of novel multi-locus genotypes, thus creating new combinations of alleles at antigen-encoding loci. It may be hypothesised that the ‘shuffling’ of alleles at different antigen-encoding loci and the generation of novel genotypes may play an important role in evasion of the host immune response. Consequently, genetic recombination may be a more important mechanism than de novo mutations for the long-term survival of *T. parva* populations.

The findings of this study have potentially important implications for vaccination of cattle. Immunity induced following infection with live *T. parva* is parasite strain-restricted and there is evidence that this strain restriction is a consequence of polymorphism in the antigens recognised by CD8^+^ T cells (Morrison et al., 2015). The current live *T. parva* vaccine includes a mixture of three parasite isolates to overcome this problem and is being used successfully in some regions (Martins et al., 2010). However, two recent studies in Kenya, one conducted in the Ol Pejeta Conservancy, have shown that vaccinated cattle introduced into pasture previously grazed predominantly by buffalo showed little or no protection against *T. parva* (Bishop et al., 2015, Sitt et al., 2015). This is consistent with the detection of extensive genotypic diversity in buffalo *T. parva* in the current study and points to the need for an alternative vaccine for use in areas where there is a high level of challenge with buffalo-derived parasites. The demonstration that some antigens that induce CD8^+^ T cell responses (e.g. Tp5. Tp6 and Tp10) have highly conserved protein sequences appears at odds with the strain-specific nature of immunity. One possibility is that the T cell responses against these antigens are inherently non-protective in cattle. Alternatively, the antigens may generally induce weak responses that are not of sufficient magnitude to confer protection. In previous studies, we have shown that cattle of MHC types known to respond to Tp1 and Tp2 generate highly dominant CD8^+^ T cell responses to these antigens, which can account for up to 70% of the total parasite-specific CD8^+^ T cell response (MacHugh et al., 2009, Connelley et al., 2011). We have proposed that the dominant nature of responses to polymorphic antigens is an important determinant of parasite strain specificity. Similar detailed information on responses to the conserved antigens is lacking. However, we have recently found that the same MHC I A10+ animals that respond to Tp2 also sometimes generate Tp10-specific CD8^+^ T cell responses, but in contrast to the Tp2 response, only 1% or less of the responding T cells recognise Tp10 (WI Morrison, unpublished data). Further work is required to determine whether this is a general feature of these conserved antigens. However, it raises the possibility that, if subunit vaccines capable of inducing protective CD8^+^ T cell responses can be developed, it may be possible to utilise conserved antigens to generate broadly cross-reactive immunity to *T. parva*.
