# Supplementary material for: Ancient diversity and geographical sub-structuring in African buffalo Theileria parva populations revealed through metagenetic analysis of antigen-encoding loci
Source: Int J Parasitol. 2018 Mar;48(3-4):287–96. doi: 10.1016/j.ijpara.2017.10.006 (PMC5854372; doi:10.1016/j.ijpara.2017.10.006)
Supplement: Supplementary Table S1 [file mmc1.docx]

**Supplementary Table S1.** Number of raw reads, number of good reads (after bioinformatic filtering), number of alleles and heterozygosity obtained for each of the animals per locus in *Theileria parva* isolated from African buffalo from the Ol Pejeta Conservancy, Kenya and the Kruger National Park, South Africa.

|  | **Ol Pejeta** | | | | |  | **Kruger National Park** | | | | | | |
| --- | --- | --- | --- | --- | --- | --- | --- | --- | --- | --- | --- | --- | --- |
| **Gene** | **Animal** | **Raw**  **reads** | **Good**  **reads** | **Alleles** | **Hz** |  | **Animal** | **Raw reads** | **Good reads** | **Alleles** | | **Hz** | |
| **Tp1** | 301 | 1213 | 1016 | 19 | 0.796 |  | SC01 | 669 | 445 | 15 | | | 0.889 |
|  | 302 | 2516 | 1978 | 16 | 0.746 |  | SC02 | 656 | 480 | 16 | | | 0.911 |
|  | 303 | 5686 | 4873 | 18 | 0.802 |  | SC03 | 591 | 398 | 7 | | | 0.771 |
|  | 304 | 25 | 6 | 1 | 0.000 |  | SC04 | 598 | 396 | 13 | | | 0.877 |
|  | 305 | 2962 | 2409 | 16 | 0.783 |  | SC05 | 2160 | 1342 | 17 | | | 0.878 |
|  | 306 | 2248 | 1934 | 16 | 0.810 |  | SC06 | 3799 | 2236 | 15 | | | 0.891 |
|  | 307 | 3274 | 2495 | 19 | 0.787 |  |  |  |  |  | | |  |
|  | 308 | 1007 | 846 | 19 | 0.839 |  |  |  |  |  | | |  |
|  | *Average* | *2366* | *1945* | *16* | *0.695* |  | *Average* | *1412* | *883* | *14* | | | *0.870* |
|  |  |  |  |  |  |  |  |  |  |  | |  | |
| **Tp2** | 301 | 404 | 322 | 26 | 0.941 |  | SC01 | 2248 | 1905 | 26 | | 0.894 | |
|  | 302 | 605 | 564 | 19 | 0.930 |  | SC02 | 377 | 289 | 18 | | 0.902 | |
|  | 303 | 158 | 92 | 10 | 0.875 |  | SC03 | 833 | 705 | 19 | | 0.885 | |
|  | 304 | 111 | 51 | 8 | 0.870 |  | SC04 | 620 | 501 | 27 | | 0.931 | |
|  | 305 | 89 | 35 | 4 | 0.700 |  | SC05 | 620 | 474 | 11 | | 0.635 | |
|  | 306 | 202 | 149 | 14 | 0.917 |  | SC06 | 683 | 583 | 26 | | 0.934 | |
|  | 307 | 182 | 103 | 10 | 0.869 |  |  |  |  |  | |  | |
|  | 308 | 246 | 181 | 16 | 0.910 |  |  |  |  |  | |  | |
|  | *Average* | *250* | *187* | *13* | *0.877* |  | *Average* | *897* | *743* | *21* | | *0.864* | |
|  |  |  |  |  |  |  |  |  |  |  |  | | |
| **Tp4** | 301 | 3013 | 10 | 2616 | 0.819 |  | SC01 | 1850 | 1678 | 23 | 0.928 | | |
|  | 302 | 963 | 14 | 799 | 0.877 |  | SC02 | 1398 | 1098 | 19 | 0.898 | | |
|  | 303 | 1192 | 13 | 860 | 0.854 |  | SC03 | 2320 | 2190 | 16 | 0.873 | | |
|  | 304 | 2198 | 18 | 1997 | 0.879 |  | SC04 | 1106 | 1010 | 22 | 0.920 | | |
|  | 305 | 2879 | 14 | 2515 | 0.845 |  | SC05 | 1010 | 841 | 25 | 0.924 | | |
|  | 306 | 1528 | 13 | 1382 | 0.863 |  | SC06 | 1876 | 1662 | 24 | 0.927 | | |
|  | 307 | 1145 | 14 | 915 | 0.815 |  |  |  |  |  |  | | |
|  | 308 | 3020 | 16 | 2302 | 0.885 |  |  |  |  |  |  | | |
|  | *Average* | *1992* | *14* | *1673* | *0.855* |  | *Average* | *1593* | *1413* | *22* | *0.912* | | |
|  |  |  |  |  |  |  |  |  |  |  |  | | |
| **Tp5** | 301 | 2491 | 2389 | 9 | 0.801 |  | SC01 | 1203 | 1092 | 15 | 0.876 | | |
|  | 302 | 2121 | 2011 | 9 | 0.806 |  | SC02 | 678 | 576 | 17 | 0.903 | | |
|  | 303 | 1941 | 1876 | 8 | 0.779 |  | SC03 | 3026 | 2827 | 15 | 0.856 | | |
|  | 304 | 1800 | 1697 | 10 | 0.804 |  | SC04 | 1703 | 1545 | 15 | 0.898 | | |
|  | 305 | 1157 | 1131 | 9 | 0.804 |  | SC05 | 1509 | 1455 | 17 | 0.900 | | |
|  | 306 | 2684 | 2572 | 7 | 0.795 |  | SC06 | 2846 | 2587 | 20 | 0.904 | | |
|  | 307 | 1674 | 1595 | 10 | 0.797 |  |  |  |  |  |  | | |
|  | 308 | 880 | 848 | 8 | 0.787 |  |  |  |  |  |  | | |
|  | *Average* | *1844* | *1765* | *9* | *0.797* |  | *Average* | *1828* | *1680* | *17* | *0.889* | | |
|  |  |  |  |  |  |  |  |  |  |  |  | | |
| **Tp6** | 301 | 1214 | 1107 | 6 | 0.523 |  | SC01 | 509 | 462 | 10 | 0.838 | | |
|  | 302 | 1047 | 967 | 6 | 0.563 |  | SC02 | 138 | 115 | 7 | 0.812 | | |
|  | 303 | 1348 | 1303 | 8 | 0.508 |  | SC03 | 1380 | 1267 | 13 | 0.849 | | |
|  | 304 | 1023 | 944 | 4 | 0.421 |  | SC04 | 711 | 628 | 18 | 0.897 | | |
|  | 305 | 1279 | 1236 | 6 | 0.529 |  | SC05 | 458 | 423 | 12 | 0.863 | | |
|  | 306 | 2029 | 1921 | 9 | 0.524 |  | SC06 | 1998 | 1845 | 16 | 0.839 | | |
|  | 307 | 1061 | 937 | 8 | 0.593 |  |  |  |  |  |  | | |
|  | 308 | 1620 | 1587 | 7 | 0.585 |  |  |  |  |  |  | | |
|  | *Average* | *1328* | *1250* | *7* | *0.531* |  | *Average* | *866* | *790* | *13* | *1* | | |
|  |  |  |  |  |  |  |  |  |  |  |  | | |
| **Tp10** | 301 | 959 | 912 | 2 | 0.011 |  | SC01 | 2455 | 2358 | 8 | 0.539 | | |
|  | 302 | 222 | 197 | 1 | 0.000 |  | SC02 | 2923 | 2811 | 10 | 0.402 | | |
|  | 303 | 1375 | 1197 | 18 | 0.790 |  | SC03 | 1641 | 1586 | 7 | 0.213 | | |
|  | 304 | 1591 | 1519 | 10 | 0.489 |  | SC04 | 189 | 143 | 1 | 0.000 | | |
|  | 305 | 199 | 158 | 5 | 0.671 |  | SC05 | 2465 | 2402 | 9 | 0.278 | | |
|  | 306 | 1295 | 1257 | 1 | 0.000 |  | SC06 | 2043 | 1725 | 11 | 0.559 | | |
|  | 307 | 228 | 204 | 1 | 0.000 |  |  |  |  |  |  | | |
|  | 308 | 2283 | 2216 | 5 | 0.299 |  |  |  |  |  |  | | |
|  | *Average* | *1019* | *958* | *5* | *0.282* |  | *Average* | *1953* | *1838* | *8* | *0.332* | | |
