# Supplementary material for: Ancient diversity and geographical sub-structuring in African buffalo Theileria parva populations revealed through metagenetic analysis of antigen-encoding loci
Source: Int J Parasitol. 2018 Mar;48(3-4):287–96. doi: 10.1016/j.ijpara.2017.10.006 (PMC5854372; doi:10.1016/j.ijpara.2017.10.006)
Supplement: Supplementary Table S2 [file mmc2.docx]

**Supplementary Table S2.** Analysis of molecular variance (AMOVA) within and between *Theileria parva* populations of African buffalo from the Ol Pejeta Conservancy, Kenya and the Kruger National Park, South Africa.

| Gene | Total estimated variance | % variance within populations | % variance between populations | PhiPT^a^ | *P* value^b^ |
| --- | --- | --- | --- | --- | --- |
| Tp1 | 0.009 | 90 | 10 | 0.104 | 0.001 |
| Tp2 | 0.162 | 97 | 3 | 0.028 | 0.001 |
| Tp4 | 0.036 | 95 | 5 | 0.053 | 0.001 |
| Tp5 | 0.010 | 97 | 3 | 0.034 | 0.068 |
| Tp6 | 0.009 | 96 | 4 | 0.042 | 0.001 |
| Tp10 | 0.014 | 93 | 7 | 0.075 | 0.032 |

^a^Measure of between-population variation which is analogous to F_ST_.

^b^The probability of between-population sequence variation contributing to overall variation based on 1,000 permutations.
