# Supplementary material for: Ancient diversity and geographical sub-structuring in African buffalo Theileria parva populations revealed through metagenetic analysis of antigen-encoding loci
Source: Int J Parasitol. 2018 Mar;48(3-4):287–96. doi: 10.1016/j.ijpara.2017.10.006 (PMC5854372; doi:10.1016/j.ijpara.2017.10.006)
Supplement: Supplementary Table S3 [file mmc3.docx]

**Supplementary Table S3.** Difference matrix based on Jaccard’s index of allele-sharing among isolates of *Theileria parva* derived from buffalo, calculated from six antigen-encoding genes.

|  | 301 | 302 | 303 | 304 | 305 | 306 | 307 | 308 | SC01 | SC02 | SC03 | SC04 | SC05 | SC06 |
| --- | --- | --- | --- | --- | --- | --- | --- | --- | --- | --- | --- | --- | --- | --- |
| 301 |  |  |  |  |  |  |  |  |  |  |  |  |  |  |
| 302 | 0.3701 |  |  |  |  |  |  |  |  |  |  |  |  |  |
| 303 | 0.5464 | 0.5653 |  |  |  |  |  |  |  |  |  |  |  |  |
| 304 | 0.6186 | 0.6169 | 0.6495 |  |  |  |  |  |  |  |  |  |  |  |
| 305 | 0.5376 | 0.5360 | 0.5425 | 0.5601 |  |  |  |  |  |  |  |  |  |  |
| 306 | 0.4348 | 0.3832 | 0.5130 | 0.6915 | 0.6326 |  |  |  |  |  |  |  |  |  |
| 307 | 0.4766 | 0.3056 | 0.5351 | 0.6222 | 0.5396 | 0.3515 |  |  |  |  |  |  |  |  |
| 308 | 0.5459 | 0.5686 | 0.5106 | 0.6591 | 0.5428 | 0.5764 | 0.5566 |  |  |  |  |  |  |  |
| SC01 | 0.9919 | 0.9910 | 0.9902 | 0.9933 | 0.9861 | 0.9889 | 0.9892 | 0.9809 |  |  |  |  |  |  |
| SC02 | 0.9400 | 0.9422 | 0.9830 | 0.9630 | 0.9697 | 0.9593 | 0.9445 | 0.9556 | 0.6568 |  |  |  |  |  |
| SC03 | 0.9545 | 0.9507 | 0.9749 | 0.9760 | 0.9731 | 0.9423 | 0.9331 | 0.9632 | 0.7181 | 0.6483 |  |  |  |  |
| SC04 | 0.9023 | 0.8223 | 0.9933 | 0.9921 | 0.9848 | 0.8126 | 0.8043 | 0.9665 | 0.6779 | 0.6924 | 0.6730 |  |  |  |
| SC05 | 0.9833 | 0.9815 | 1.0000 | 1.0000 | 1.0000 | 0.9815 | 0.9771 | 1.0000 | 0.7064 | 0.6255 | 0.6290 | 0.7263 |  |  |
| SC06 | 0.9653 | 0.9701 | 0.9746 | 0.9715 | 0.9677 | 0.9718 | 0.9632 | 0.9583 | 0.6341 | 0.6040 | 0.6891 | 0.6966 | 0.6225 |  |
