# Supplementary material for: Ancient diversity and geographical sub-structuring in African buffalo Theileria parva populations revealed through metagenetic analysis of antigen-encoding loci
Source: Int J Parasitol. 2018 Mar;48(3-4):287–96. doi: 10.1016/j.ijpara.2017.10.006 (PMC5854372; doi:10.1016/j.ijpara.2017.10.006)
Supplement: Supplementary Table S4 [file mmc4.docx]

|  | Total number of  polymorphic residues | Shared polymorphic residues | Polymorphic residues  ‘private’ to Ol Pejeta | Polymorphic residues ‘private’ to Kruger NP |
| --- | --- | --- | --- | --- |
| Tp1 | 35 | 9 | 15 | 11 |
| Tp2 | 168 | 149 | 9 | 10 |
| Tp4 | 114 | 67 | 9 | 36 |
| Tp5 | 29 | 13 | 3 | 13 |
| Tp6 | 28 | 12 | 3 | 13 |
| Tp10 | 30 | 15 | 7 | 8 |

**Supplementary Table S4.** Proportions of polymorphic nucleotide residues of six *Theileria parva* antigen-encoding gene segments from buffalo unique to the Ol Pejeta Conservancy (Kenya) or Kruger National Park (South Africa) sequences.
